# Race-Specific and Race-Neutral Equations for Lung Function and Asthma Diagnosis in Black Children

**DOI:** 10.1001/jamanetworkopen.2024.62176

**Published:** 2025-02-28

**Authors:** Wan Chi Chang, Jeffrey W. Burkle, Lindsey R. Williams, Mindy K. Hammonds, Farida A. Weheba, Latha Satish, Lisa J. Martin, Theresa W. Guilbert, Michael G. Sherenian, Tesfaye B. Mersha, Jocelyn M. Biagini, Gurjit K. Khurana Hershey

**Affiliations:** 1 Division of Asthma Research, Cincinnati Children’s Hospital Medical Center, Cincinnati, Ohio; 2 Department of Pediatrics, University of Cincinnati College of Medicine, Cincinnati, Ohio; 3 Division of Human Genetics, Cincinnati Children’s Hospital Medical Center, Cincinnati, Ohio; 4 Division of Pulmonary Medicine—Clinical, Cincinnati Children’s Hospital Medical Center, Cincinnati, Ohio

## Abstract

**Importance:**

Use of the race-neutral Global Lung Initiative (GLI) equation has been shown to generate decreased lung function measures in Black children and adults. The effect on asthma detection and diagnosis in children is unknown.

**Objective:**

To compare the use of race-specific vs race-neutral equations on subsequent asthma diagnosis in children.

**Design, Setting, and Participants:**

The Childhood Asthma Management Program (CAMP, 1991-2012), the Cincinnati Childhood Allergy and Air Pollution Study (CCAAPS, 2001-2010), and the Mechanisms of Progression from Atopic Dermatitis to Asthma (MPAACH, 2016-2024) cohorts were included in this cohort study. Children in the CAMP cohort were aged 5 to 12 years with mild to moderate asthma. The CCAAPS and MPAACH cohorts included infants from atopic parents and children aged 0 to 2 years with atopic dermatitis, respectively. Data were analyzed from November 2023 to May 2024.

**Exposures:**

Race-specific vs race-neutral GLI equations to define lung function.

**Main Outcomes and Measures:**

Percent predicted values of forced expiratory volume in 1 second (FEV_1_), forced vital capacity (FVC), asthma or asthma symptoms, and eligibility for reversibility testing were determined.

**Results:**

Among 1533 children, there were 849 CAMP (median [IQR] age, 8.7 [7.1-10.6] years; 138 [16%] Black, 711 [84%] White, and 498 [59%] male participants), 578 CCAAPS (median [IQR] age, 6.9 [6.7-7.0]; 115 [20%] Black, 463 [80%] White, and 315 [55%] male participants) and 106 MPAACH (median [IQR] age, 7.4 [7.1-7.8] years; 62 [58%] Black, 44 [42%] White, and 62 [58%] male participants). The median (IQR) percent predicted FEV_1_ in Black children decreased by 11.9 percentage points (pp) (10.4-13.1 pp) in CAMP, 13.5% pp (11.8-14.6 pp) in CCAAPS, and 13.2 pp (11.6-14.6 pp) in MPAACH compared with the race-specific equation. The race-specific equation failed to detect reduced percent predicted FEV_1_ in 12 of 22 Black children in CCAAPS with asthma symptoms (55%) and 5 of 15 Black children in MPAACH with asthma (41%). In CCAAPS, children with less than 90% predicted FEV_1_ based on race-specific equations were eligible for postreversibility testing to objectively diagnose asthma. When this asthma diagnostic algorithm was applied, 16 of 36 Black children in CCAAPS (44%) and 6 of 16 Black children in MPAACH (38%) who were not eligible for reversibility testing based on the race-specific equation became eligible with a less than 90% predicted FEV_1_ based on the race-neutral equation.

**Conclusions and Relevance:**

In this cohort study of 1533 children, the use of the race-neutral equation improved the detection of asthma in children. These results support the universal use of the race-neutral equation to improve asthma detection in children and help guide medical practice toward alleviating asthma-related health disparities.

## Key Points

**Question:**

Does excluding race from Global Lung Initiative reference equations change pediatric asthma detection?

**Findings:**

In this cohort study of 1533 children who underwent spirometry, percent predicted forced expiratory volume in 1 second and forced vital capacity for Black children were significantly lower using race-neutral equations compared with race-specific equations. Furthermore, the race-neutral equation identified 2- to 4-fold more Black children with asthma or asthma symptoms as having reduced lung function, increasing the likelihood of further testing and asthma detection.

**Meaning:**

These findings have significant clinical implications and further support the universal use of race-neutral equations to increase the likelihood of identifying reduced lung function and improving the detection of asthma, particularly in Black children, promoting health equity.

## Teaser

This cohort study evaluates the use of race-specific vs race-neutral equations on subsequent asthma diagnosis in children.

## Introduction

Health disparities in allergic disorders have been documented for decades,^1^ persisting with age even when controlling for socioeconomic status.^2^ Black children have a higher prevalence of asthma and increased rates of asthma morbidity than White children, persisting across childhood.^3^ In children, asthma is diagnosed according to the Guidelines for the Diagnosis and Management of Asthma: Expert Panel Report 3,^4,5^ guided by measures of forced expiratory volume in 1 second (FEV_1_) and forced vital capacity (FVC).

As FEV_1_ is a function of age, sex, and body size, standardization is required; thus, reference equations are used to determine percent predicted FEV_1_ and FVC. These equations are historically adjusted for race, based upon flawed studies suggesting that White people have “naturally higher” lung capacity compared with other races.^6^ The current standard equations developed in 2012 by the Global Lung Function Initiative (GLI) adjust for age, sex, height, and race (White, Black, North East Asian, South East Asian, and Other).^7^

These equations have been built into US spirometers and apply a 10% to 15% adjustment for individuals labeled Black and 4% to 6% for those labeled as Asian,^6^ leading to an underestimation of likely abnormal results in Black children^3,8,9,10^ and propagating structural racism into respiratory medicine. In clinical decision-making for asthma, the use of race-specific equations could lead to underdiagnosis of asthma in Black children because it fails to recognize that race itself is a social construct^11^ confounded with socioeconomic factors.^12^

Recognizing that the use of race-specific equations do not account for the deleterious effects of social and environmental exposures that disproportionately affect marginalized communities, the GLI 2022 neutral or global equation was derived as a single race-neutral reference equation that does not require any selection of race.^9^ This study and others show that when the race-neutral equation is used in place of the race-specific equation, Black individuals are classified as having more abnormal lung function, with White individuals having opposing changes.^8,10,13,14,15^ It is estimated that the use of the race-neutral equation may reclassify abnormal lung function for 12.5 million Americans, increasing 141% for Black persons and decreasing 69% among White persons.^13^ More recent studies in the US also show that the use of race-specific equations for lung function may inaccurately estimate impairments in marginalized, vulnerable children and adults in relation to clinical end points.^9^ Race-specific equations underestimate mortality risk in adults^16^ and severity of chronic obstructive pulmonary disease^17^ in adult Black individuals, but studies in asthma are lacking.

In the present analysis, we sought to examine the percent predicted FEV_1_ and FVC assigned through race-specific and race-neutral equations, determine the changes in identification of reduced FEV_1_ in 3 asthma and high-risk cohorts, and determine the ability of these equations to detect asthma in Cincinnati Childhood Allergy and Air Pollution Study (CCAAPS) and Mechanisms of Progression from Atopic Dermatitis to Asthma (MPAACH).

## Methods

### Study Participants

The Childhood Asthma Management Program (CAMP) recruited children aged 5 to 12 years with chronic mild to moderate asthma and current asthma symptoms with sensitivity to methacholine from 1993 to 1995, with a follow-up period of 5 to 6 years.^18^ CCAAPS is a longitudinal high-risk birth cohort that recruited infants with atopic parent(s) between 2001 and 2003. Participants were followed up until the age of 7.^19^ MPAACH is a prospective longitudinal early-life cohort of children aged 0 to 2 years with atopic dermatitis that has been previously described.^20,21^ Children in MPAACH were recruited between 2016 and 2024 and followed up for 5 years across 5 annual visits. In CAMP, each parent or guardian signed a consent statement, and participants signed an assent statement approved by each clinical center’s institutional review board. In CCAAPS and MPAACH, the legal guardian provided informed consent approved by the institutional review board at the University of Cincinnati and Cincinnati Children’s Hospital Medical Center, respectively. All cohorts as well as the present adhered to the Strengthening the Reporting of Observational Studies in Epidemiology (STROBE) reporting guidelines. A flow diagram of participants included in this analysis is in eFigure 1 in Supplement 1. Additional details on study participants, pulmonary function testing, self-reported race and ancestry, reference equations, and statistical analyses can be found in the eTable and eMethods in Supplement 1.

### Pulmonary Function Testing

In CAMP, spirometry was performed according to American Thoracic Society (ATS) criteria^22,23^ in children aged 5 to 12 years. In CCAAPS and MPAACH, spirometry was performed in children aged 7 or 8 years, also according to the ATS criteria. Spirometry results were reviewed by physicians, and trials with the best FEV_1_ and FVC were determined. In CCAAPS, those reporting asthma symptoms, an exhaled nitric oxide concentration greater than 20 parts per billion, or a predicted FEV_1_ less than 90% and/or an FEV_1_/FVC less than the lower limit of normal underwent reversibility testing. In this study, a predicted FEV_1_ less than 90% was considered reduced and used to characterize potential obstructive lung function.

### Asthma Symptoms and Diagnosis

All CAMP participants had current chronic mild to moderate asthma. Data on wheezing episodes, hospital admission for wheezing or asthma, physician or emergency care visits for asthma, and usage of an asthma medication were also collected. In CCAAPS, at age 7, asthma symptoms including wheezing in the past 12 months, hospital admission for wheezing or asthma, physician or emergency care visits for asthma, and usage of an asthma medication or treatment were reported by parents via questionnaire. In MPAACH, asthma symptoms including wheezing in the past 12 months, hospitalization for wheezing or asthma, 2 or more physician or emergency care visits for asthma, and 6 or more months’ usage of an asthma controller were reported by parents via questionnaire. The algorithms used to objectively diagnose asthma in CCAAPS and MPAACH can be found in the eMethods in Supplement 1.

### Statistical Analyses

Data were assessed and analyzed from November 2023 to May 2024. Wilcoxon rank-sum tests were used to compare differences in the percentage of predicted lung function values between Black and White children. To determine the differences between each equation in the number of children with asthma symptoms identified, McNemar tests were used. Statistical significance was defined as a *P* value less than .05. Statistical analyses were performed in R version 4.1.0 (R Project for Statistical Computing).

## Results

### Participant Characteristics

At the time of analysis, there were 849 CAMP (median [IQR] age, 8.7 [7.1-10.6] years; 138 [16%] Black, 711 [84%] White, and 498 [59%] male participants), 578 CCAAPS (median [IQR] age, 6.9 [6.7-7.0]; 115 [20%] Black, 463 [80%] White, and 315 [55%] male participants) and 106 MPAACH (median [IQR] age, 7.4 [7.1-7.8] years; 62 [58%] Black, 44 [42%] White, and 62 [58%] male participants). Within CCAAPS, Black children were more likely to have at least 2 physician and/or emergency department visits for asthma (15 [13%] Black vs 30 [6%] White participants) and to be diagnosed with asthma by a physician (29 [25%] Black vs 56 [12%] White participants) compared with White children. There were no significant differences in age, sex, wheezing history, hospitalization for wheezing or asthma, or controller usage in the last 12 months (Table).

**Table.** Characteristics of Study Participants

Characteristic	Participants, No. (%)			*P* value
	Overall	Black^a^	White^a^	
CAMP				
Total No.	849 (100)	138 (16)	711 (84)	
Age, y, median (IQR)	8.7 (7.1-10.6)	9.1 (7.5-10.6)	8.7 (7.1-10.6)	.33
Sex				
Male	498 (59)	82 (59)	416 (59)	.92
Female	351 (41)	56 (41)	295 (41)	
Asthma symptoms				
Wheezing	795 (94)	133 (96)	662 (93)	.18
Hospitalization for wheezing or asthma	53 (6)	10 (7)	43 (6)	.57
≥2 Physician or ED visits for asthma	700 (82)	114 (83)	586 (82)	.99
Asthma controller use^b^	535 (63)	91 (66)	444 (62)	.50
CCAAPS				
Total No.	578 (100)	115 (20)	463 (80)	
Age, y, median (IQR)	6.9 (6.7-7.0)	6.9 (6.7-7.0)	6.9 (6.7-7.0)	.36
Sex				
Male	315 (55)	64 (56)	251 (54)	.83
Female	263 (45)	51 (44)	212 (46)	
Asthma symptoms				
Wheezing	90 (16)	22 (19)	68 (15)	.25
Hospitalization for wheezing or asthma	2 (0.3)	1 (0.9)	1 (0.2)	.36
≥2 Physician or ED visits for asthma	45 (8)	15 (13)	30 (6)	.03
Asthma controller use^b^	67 (12)	17 (15)	50 (11)	.25
Physician diagnosed asthma	85 (15)	29 (25)	56 (12)	.001
MPAACH				
Total No.	106 (100)	62 (58)	44 (42)	
Age, y, median (IQR)	7.4 (7.1-7.8)	7.5 (7.2-7.8)	7.4 (7.1-7.8)	.19
Sex				
Male	62 (58)	33 (53)	29 (66)	.23
Female	44 (42)	29 (47)	15 (34)	
Asthma symptoms				
Wheezing	35 (33)	22 (35)	13 (30)	.54
Hospitalization for wheezing or asthma	4 (4)	4 (6)	0 (0)	.14
≥2 Physician or ED visits for asthma	12 (11)	9 (15)	3 (7)	.35
Asthma controller use				
<6 mo	10 (9)	6 (10)	4 (9)	.95
≥6 mo	22 (21)	12 (19)	10 (23)	
Physician diagnosed asthma	32 (30)	20 (32)	12 (27)	.68

Abbreviations: CAMP, Childhood Asthma Management Program; CCAAPS, Cincinnati Childhood Allergy and Air Pollution; ED, emergency department; MPAACH, Mechanisms of Progression from Atopic Dermatitis to Asthma.

^a^ Comparisons between white and black children were done using Wilcoxon rank-sum and Fisher exact tests for continuous and categorical variables, respectively.

^b^ Asthma controller use in CAMP and CCAAPS was recorded as any usage of asthma controller or medication.

### Reduced Percent Predicted FEV_1_ and FVC Values in Black Children

**Figure 1.**
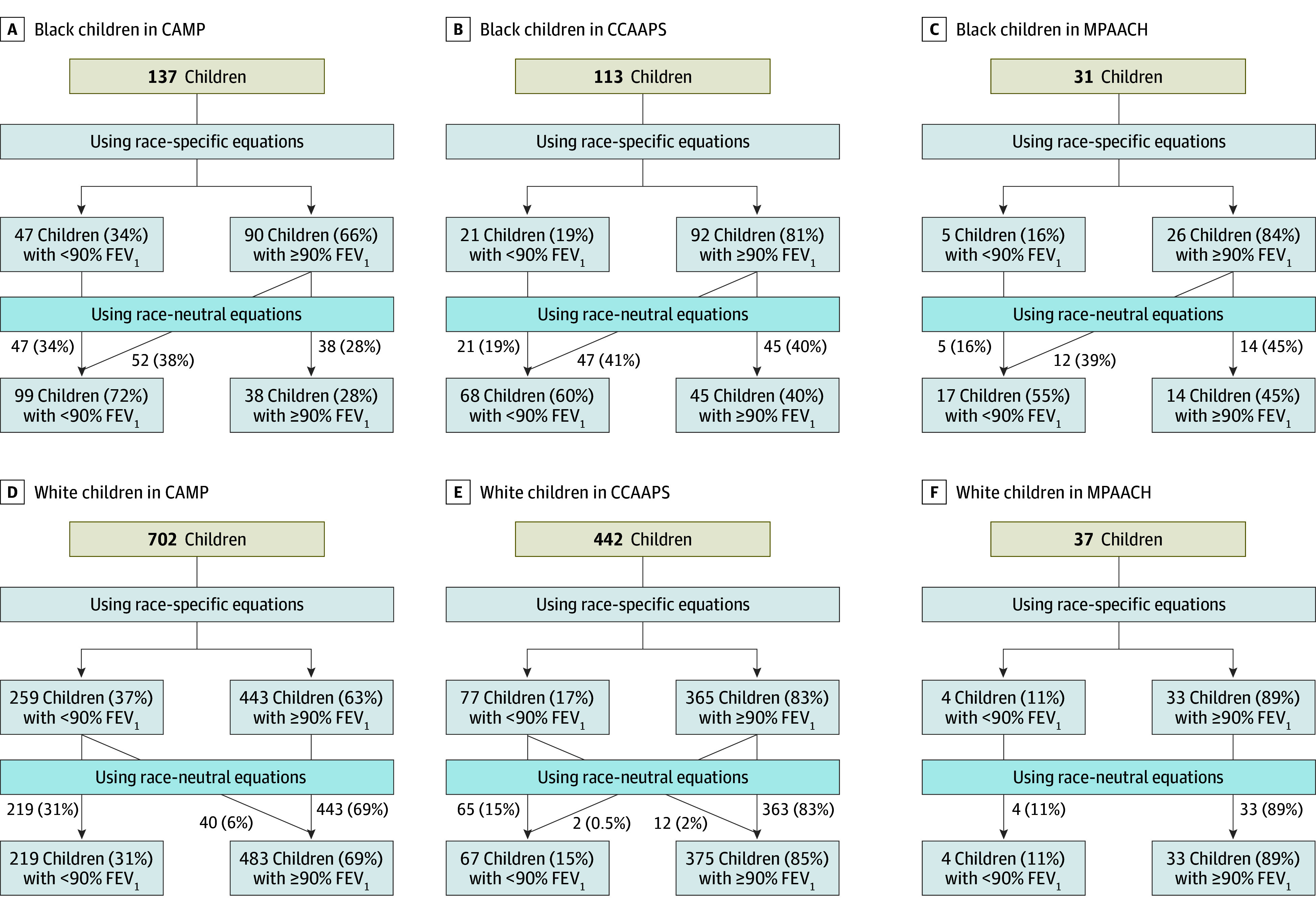
Change of Classification in Forced Expiratory Volume in 1 Second (FEV_1_) Using Race-Specific vs Race-Neutral Equations Black and White children in CAMP, CCAAPS, and MPAACH who were reclassified as having reduced FEV_1_ after moving from race-specific to race-neutral equations. Reduced FEV_1_ was defined as less than 90% of predicted values. CAMP indicates Childhood Asthma Management Program; CCAAPS, Cincinnati Childhood Allergy and Air Pollution; MPAACH, Mechanisms of Progression from Atopic Dermatitis to Asthma.

839 children in CAMP, 555 children in CCAAPS, and 68 children in MPAACH had acceptable and reproducible spirometry data and were included in subsequent analyses. Overall, 111 of 281 Black children across all cohorts (39%) changed from normal (≥90% predicted) to reduced (<90% predicted) FEV_1_ when the race-neutral equation was applied (38% in CAMP, 41% in CCAAPS, and 39% in MPAACH) (Figure 1A-C). In contrast, 6% of CAMP and 2% of CCAAPS White children changed from reduced to normal when using the race-neutral equation, and no change was observed in White children in MPAACH (Figure 1D-F).

**Figure 2.**
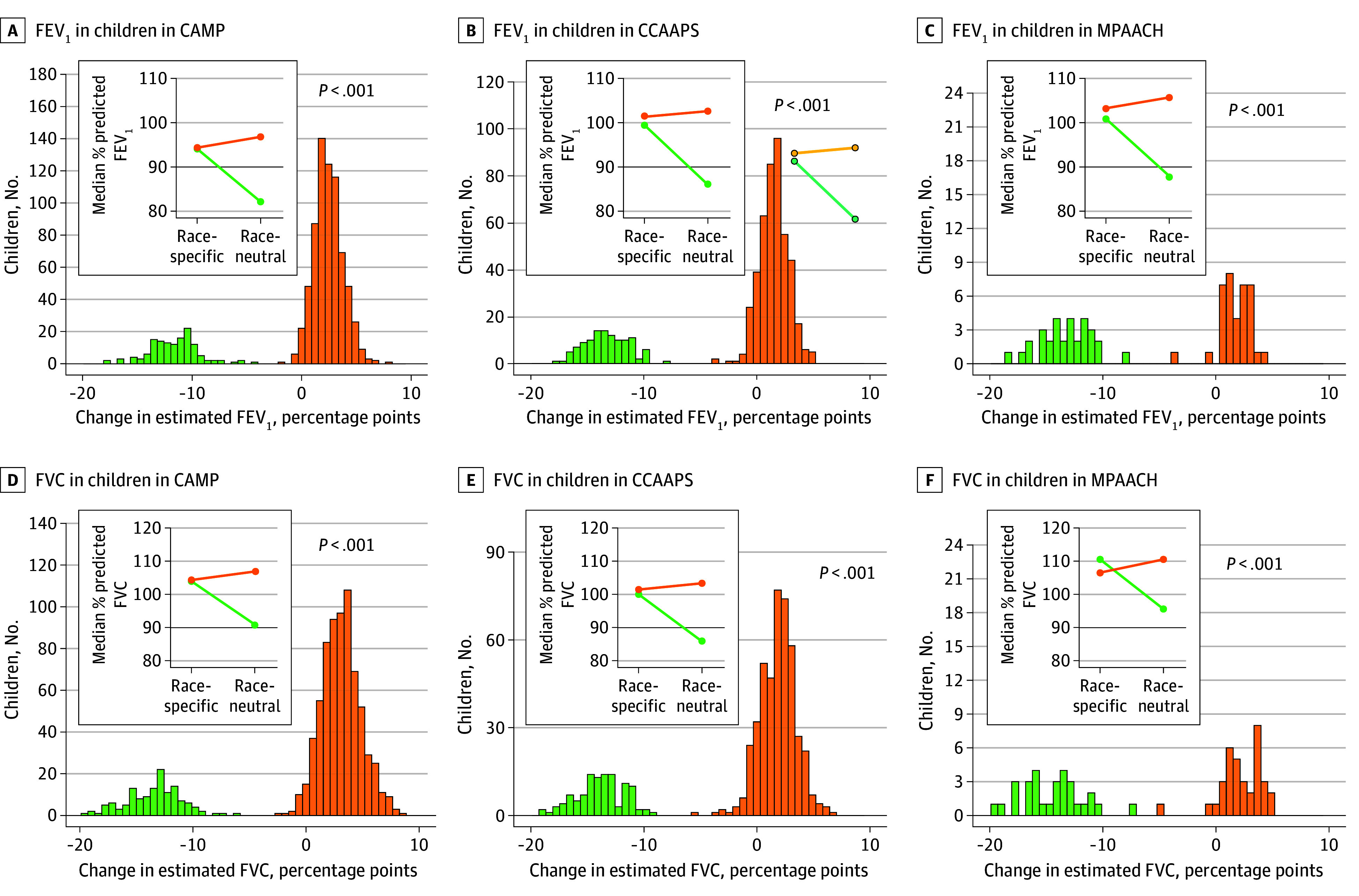
Changes in Percent Predicted Forced Expiratory Volume in 1 Second (FEV_1_) and Forced Vital Capacity (FVC) Using Race-Specific vs Race-Neutral Approaches in Black and White Children Differences in percent predicted FEV_1_ and FVC among children in CAMP (137 Black and 702 White), children in CCAAPS (113 Black and 442 White), and children in MPAACH (31 Black and 37 White) when applying race-specific and race-neutral equations. An increase in percent predicted values represented an improvement in apparent pulmonary function, whereas a decrease indicated a decline in apparent pulmonary function. Changes in the percent predicted values were calculated as the percentage predicted derived from race-neutral equation minus the percentage predicted derived from race-specific equation. Differences between races were compared using Wilcoxon rank-sum tests. The green and orange colors represent Black and White children, respectively. CAMP indicates Childhood Asthma Management Program; CCAAPS, Cincinnati Childhood Allergy and Air Pollution; MPAACH, Mechanisms of Progression from Atopic Dermatitis to Asthma.

Changes in the percent predicted values of lung function measures are presented in Figure 2. In all cohorts, substituting the race-specific equation with the race-neutral equation resulted in a significant decrease in the percent predicted FEV_1_ (Figure 2A-C) and FVC in Black children (Figure 2D-F), while an increase was observed in White children. The median percent predicted FEV_1_ in Black children decreased by 12.9 percentage points (pp) (10.9 to 13.9 pp; CAMP: −11.9 pp [−10.4 to −13.1 pp]; CCAAPS: −13.5 pp [−11.8 to −14.6 pp]; MPAACH: −13.2 pp [−11.6 to −14.6 pp]) when the race-neutral equation was used, resulting in the median FEV_1_ falling within the reduced range (Figure 2A-C). Similarly, the median (IQR) predicted FVC of Black children was 14.3 pp (12.2 to 15.2 pp) lower (CAMP: −13.4 pp [−11.9 to −15.1 pp]; CCAAPS: −14.1 pp [−12.4 to −15.1 pp]; MPAACH: −15.4 pp [−13.1 to −16.4% pp]) using the race-neutral equation (Figure 2D-F). Changes in percent predicted FEV_1_/FVC between Black and White children were minimal (eFigure 2 in Supplement 1).

The distribution of lung function measures for individual participants and comparisons between Black and White children are presented in eFigures 3, 4, and 5 in Supplement 1. Since race was defined by self-report, we also evaluated percent predicted FEV_1_ and FVC by percentage genetic African ancestry, and the results were the same as with self-reported race (eFigure 6 in Supplement 1). Collectively, these findings suggest that the race-specific equation overestimates lung function in Black children, which may lead to underdiagnosis of asthma in this susceptible group.

### Increase in Black Children With Asthma and Asthma-Related Symptoms Having <90% Predicted FEV_1_

**Figure 3.**
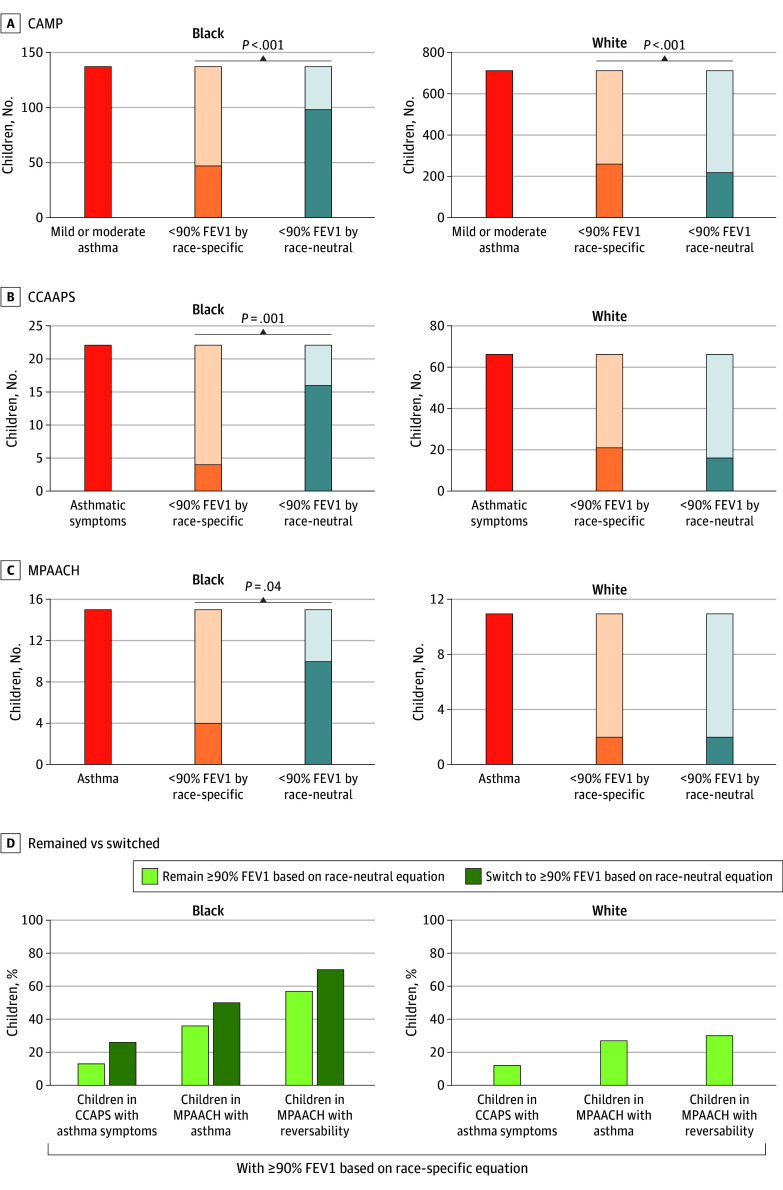
The Number of Children With Asthma, Asthma Symptoms, and Reversibility, Assessed by the 2 Global Lung Initiative Equations In CAMP, CCAAPS, and MPAACH, the number of children with asthma or asthma-related symptoms when labeled as having reduced FEV_1_ values by each equation. McNemar tests were used to compare differences in the proportion of identified children with symptoms between the 2 equations. Reduced FEV_1_ values were assessed using race-specific (orange) and race-neutral (blue) equations and defined as less than 90% of predicted FEV_1_. D, Among children with a predicted FEV_1_ of 90% or higher using race-specific equations, the prevalence of asthma diagnosis, clinical symptoms of asthma, and reversibility were compared between the 2 subgroups: (1) remained with a 90% or higher predicted FEV_1_ based on race-neutral equations (light green bars) or (2) switched to a 90% or higher predicted FEV_1_ when based on race-neutral equations (dark green bars). CAMP indicates Childhood Asthma Management Program; CCAAPS, Cincinnati Childhood Allergy and Air Pollution; FEV_1_, forced expiratory volume in 1 second; MPAACH, Mechanisms of Progression from Atopic Dermatitis to Asthma.

We next sought to determine if the race-neutral equation identified more children with asthma and asthma-related symptoms as having reduced FEV_1_ compared with the race-specific equation. At the time when the CAMP and CCAAPS studies were conducted, only the race-specific equation was available. Application of the race-neutral reference equation to the pulmonary function testing (PFT) data detected reduced FEV_1_ in 52 (38%) Black children in CAMP with asthma that were previously identified as having normal FEV_1_. This represents a 2.1-fold increase in the number of Black children with asthma symptoms who were identified as having a reduced percent predicted FEV_1_ compared with the race-specific equation (χ^2^_1_ = 50.0; *P* < .001) (Figure 3A). In CCAAPS, the race-neutral equation identified 16 out of 22 (73%) Black children with asthma symptoms as having reduced lung function, 4 times higher than the 4 (18%) identified using the race-specific equation (χ^2^_1_ = 10.1; *P* = .001) (Figure 3B). Since all children in MPAACH undergo reversibility testing at ages 7 to 8 years, we have asthma diagnoses available for children that have completed testing. There were 15 Black children defined as having asthma in MPAACH based on symptoms and/or reversibility. The race-specific equation detected 4 out of 15 (26%) Black children with asthma as having reduced FEV_1_, whereas the race-neutral equation detected 10 (67%), a 2.5-fold increase (χ^2^_1_ = 4.2; *P* = .04) (Figure 3C). The race-specific equation failed to detect reduced percent predicted FEV_1_ in 12 of 22 of Black children in CCAAPS with asthma symptoms (55%) and 6 of 15 Black children in MPAACH with asthma (41%). In contrast, use of the race-neutral equation resulted in an increase in FEV_1_ in White children in CAMP with asthma (40 of 702 children [6%]) (Figure 3A) and White children in CCAAPS with asthma symptoms (5 of 66 children [8%]) (Figure 3B) compared with the race-specific equation. The number of White children with asthma in the reduced FEV_1_ group remained constant across both GLI equations in MPAACH (Figure 3C).

To further assess the clinical changes associated with the race-neutral equation, we examined its ability to improve asthma detection in Black children. We focused on children in MPAACH and CCAAPS who exhibited normal spirometry (≥90% predicted FEV_1_) using race-specific equations but were identified as having less than 90% predicted FEV_1_ by the race-neutral equation and compared them with children who had 90% or higher predicted FEV_1_ using either equation. Among the group who exhibited normal spirometry (≥90% predicted FEV_1_) using race-specific equations but were identified as having less than 90% predicted FEV_1_ by the race-neutral equation, there were 2-fold more Black children in CCAAPS with asthma symptoms. Similarly, there were 1.4-fold more Black children in MPAACH with asthma and 1.2-fold more Black children in MPAACH who exhibited reversibility compared with those who had 90% or greater predicted FEV_1_ using either equation (Figure 3D; eFigure 7 in Supplement 1). Given the small sample size in this restricted analysis, these results did not reach statistical significance but support that Black children with asthma or asthma symptoms are likely underdiagnosed when the race-specific equation is used. In contrast, no additional White children with asthma, asthma symptoms, or reversibility were detected when the race-neutral equations were applied (Figure 3D; eFigure 7 in Supplement 1).

### Lack of Follow-Up for Potential Asthma Diagnosis

**Figure 4.**
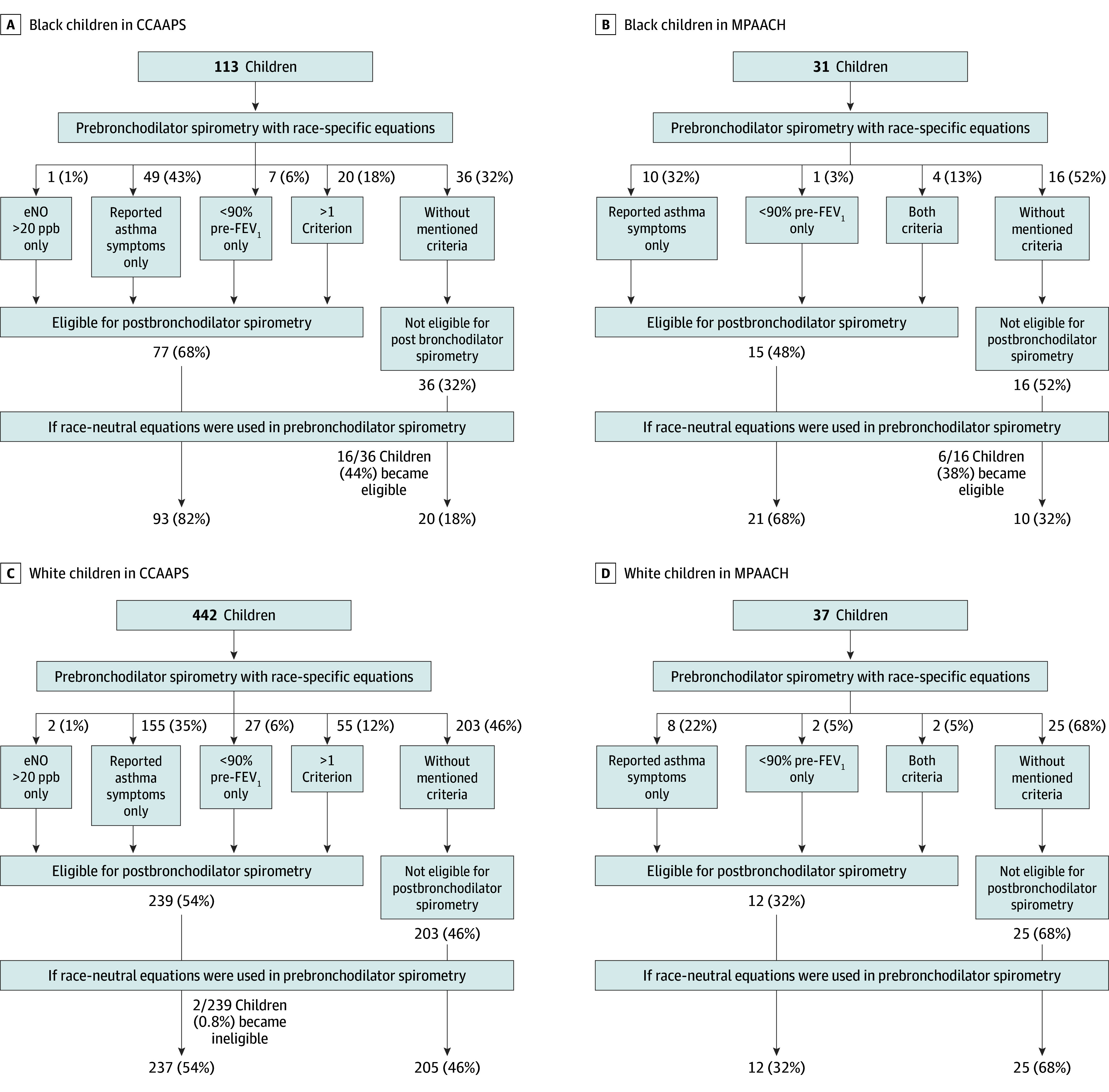
Eligibility of Postbronchodilator Spirometry for Cincinnati Childhood Allergy and Air Pollution (CCAAPS) and Mechanisms of Progression from Atopic Dermatitis to Asthma (MPAACH) Children Using CCAAPS Algorithm Based on the CCAAPS algorithm, the eligibility of postbronchodilator spirometry testing using race-specific or race-neutral equations in Black children in CCAAPS, Black children in MPAACH, White children in CCAAPS, and White children in MPAACH. Exhaled nitric oxide test was administered in CCAAPS only. eNO indicates exhaled nitric oxide; FEV_1_, forced expiratory volume in 1 second; ppb, parts per billion.

In CCAAPS, all children performed baseline PFT testing and were objectively diagnosed with asthma according to a published algorithm.^24^ Among other criteria (see eMethods in Supplement 1), children with less than 90% predicted FEV_1_ were sent for reversibility testing.^24^ Using the race-specific equation applied by the original cohort in 2008 to 2010, 77 of 113 Black children were sent for reversibility testing (Figure 4A). When we applied the race-neutral equation to the 36 children who were not sent for reversibility testing, 16 of the 36 (44%) were identified as having less than 90% predicted FEV_1_, and therefore should have been sent for reversibility testing (Figure 4A). When we applied the CCAAPS algorithm to MPAACH, 16 of the 31 Black children were not eligible for posttesting using the race-specific equation. However, when the race-neutral equation was applied, 6 of 16 of these children (38%) had less than 90% predicted FEV_1_ (Figure 4B). In contrast, there was minimal to no change in posttesting eligibility in White children in CCAAPS and MPAACH (Figure 4C-D). Thus, use of the race-specific equation led to 16 of 36 Black children in CCAAPS (44%) and 6 of 16 of Black children in MPAACH (38%) not being sent for further testing to determine if they had an asthma diagnosis.

## Discussion

This study found that the 2012 GLI race-specific equation failed to detect reduced lung function in 39% of Black children participating in 3 large cohorts compared with the 2022 GLI race-neutral equation. We observed that the race-specific equation produced, on average, 12.9% lower predicted FEV_1_ and 14.3% lower FVC compared with the race-neutral equation in Black children, which is similar in magnitude compared with other studies.^10,25^ When we tested the ability of the 2 equations to detect potential asthma and asthma symptoms in children in CCAAPS and MPAACH, we observed that the race-neutral equation labeled 2.5- to 4-fold more Black children with asthma symptoms as having reduced lung function, with only a slight change for White children. Furthermore, when we restricted the analysis to only those children with greater than 90% FEV_1_ using the race-specific equation and then applied the race-neutral equation, 1.4- to 2-fold more Black children with asthma and asthma symptoms were detected. Collectively, our data support evidence that Black children with asthma or asthma symptoms are likely underdiagnosed when the race-specific equation is used.

While the use of the race-neutral equation results in decreased percent predicted FEV_1_, to our knowledge, these equations have not been compared for the purposes of detecting asthma in children. This is clinically relevant as initiation of asthma treatment may be delayed in these children, and children with current asthma symptoms and an FEV_1_ less than 90% would be more likely to undergo follow-up with postbronchodilator testing for potential asthma diagnosis. The use of the race-neutral equation moves respiratory science closer to removing race, reducing structural racism, and demoting health disparities that have plagued asthma research and treatment for decades.

We and others observed Black children to have significantly decreased percent predicted FEV_1_ and FVC when using the race-neutral equation compared with the race-specific equation.^9,10,13,14,15^ Use of the race-neutral equation has been shown to result in a significant increase in the number of Black individuals with abnormal lung function, along with a significant increase in the severity of impairment,^8,13,15^ further promoting health disparities.^10^ A previous study^26^ showed that the use of the race-specific equation in Black children with persistent asthma results in a higher number being inappropriately labeled as controlled, which could also increase health disparities and increase the risk of long-term negative effects on lung health. In this analysis, we advanced these findings to show that if the race-neutral equation is applied to Black children with FEV_1_ greater than 90% based on the race-specific equation, more Black children with known asthma or asthma symptoms are detected. This has significant clinical implications, as a child presenting with asthma symptoms but a normal FEV_1_ and FVC based on race-specific values would be much less likely to be further evaluated for asthma than a child presenting with symptoms and a reduced PFT, leading to underdiagnosis of asthma. This is clinically relevant as race-specific equations fail to detect reduced FEV_1_ in over a third to half of Black asthmatic children, and initiation of asthma treatment may be delayed in these children.

Clinically, PFTs are often used to objectively define asthma. In CCAAPS, PFTs were conducted with the race-specific equations for diagnosing asthma in the original cohort.^24^ As the data herein show, the race-neutral equation resulted in 44% of Black children in CCAAPS who were not sent for reversibility testing having a percent predicted FEV_1_ less than 90%, so asthma was likely underdiagnosed in this already susceptible population. Children who are not identified as having reduced lung function due to the use of race-specific equations may be underdiagnosed. Furthermore, initiation of treatment may be delayed, perpetuating the racial disparities in asthma prevalence and causing further damage to the lungs of Black children.^27^

### Strengths and Limitations

Our study had multiple strengths, including the inclusion of 3 diverse cohorts and the clinical evaluation of asthma symptoms. Our cohorts included 16% to 58% Black children, which enabled the evaluation of spirometry results stratified by race. The 3 cohorts included a mild to moderate asthma cohort, a high-risk birth cohort, and an eczema cohort, highlighting the generalizability of our findings. CAMP is a clinical trial, and CCAAPS and MPAACH are longitudinal prospective observational cohorts, which enabled us to collect extensive phenotypic information that is not accessible in datasets extracted from an electronic medical record. There were also limitations. Our data only included Black and White individuals, limiting generalizability to other races and ethnicities. While the use of the race-neutral equation may lead to earlier detection and treatment for children with uncontrolled asthma, it could potentially lead to over-treatment in well-controlled children with asthma. The MPAACH cohort could have been subject to selection bias since all children had atopic dermatitis and therefore were at higher risk of asthma. Asthma symptoms were collected through parent-report and could introduce measurement error. Additionally, the PFTs were collected during a single visit, so future PFT testing may be needed to assess changes over time.

## Conclusions

In this cohort study of children, our findings support that use of the race-specific equation promotes health disparities with respect to lung function and results in missed asthma diagnoses in our most vulnerable populations. It also continues to embed structural racism in clinical practice where clinicians and researchers may be unaware and thus unknowingly propagate structural racism and health disparities. The use of a race-neutral equation promotes the identification of deficits in lung function that the race-specific equation fails to detect, particularly in Black children. These results provide further support to the 2023 recommendation from the ATS to adopt a race-neutral approach to PFT interpretation in all PFT laboratories.^28^ A shift to universal use of the race-neutral equation will likely improve the detection of asthma, decrease the risk of labeling uncontrolled asthma as controlled, and keep moving science away from outdated racist practices and toward alleviating asthma-related health disparities, promoting health equality.

## Notes

Supplement 1.
eMethods.

eReferences.
**eTable. **Model structures of 2 GLI equations **eFigure 1.** CONSORT diagram of the participants included in the analyses **eFigure 2.** Changes in FEV_1_/FVC using race-specific vs race-neutral equations **eFigure 3.** Estimated values of lung function measures using race-specific and race-neutral equations in CAMP **eFigure 4.** Estimated values of lung function measures using race-specific and race-neutral equations in CCAAPS **eFigure 5. **Estimated values of lung function measures using race-specific and race-neutral equations in MPAACH **eFigure 6.** Associations between estimated lung function measures by GLI equations and African ancestry **eFigure 7.** Prevalence of asthma, asthma symptoms, and reversibility in CCAAPS and MPAACH children by 2 GLI equations

Supplement 2.
Data Sharing Statement
