# Supplementary material for: Race-Specific and Race-Neutral Equations for Lung Function and Asthma Diagnosis in Black Children
Source: JAMA Netw Open. 2025 Feb 28;8(2):e2462176. doi: 10.1001/jamanetworkopen.2024.62176 (PMC11871546; doi:10.1001/jamanetworkopen.2024.62176)
Supplement: Supplement 1. — eMethods. eReferences. eTable. Model structures of 2 GLI equations eFigure 1. CONSORT diagram of the participants included in the analyses eFigure 2. Changes in FEV1/FVC using race-specific vs race-neutral equations eFigure 3. Estimated values of lung function measures using race-specific and race-neutral equations in CAMP eFigure 4. Estimated values of lung function measures using race-specific and race-neutral equations in CCAAPS eFigure 5. Estimated values of lung function measures using race-specific and race-neutral equations in MPAACH eFigure 6. Associations between estimated lung function measures by GLI equations and African ancestry eFigure 7. Prevalence of asthma, asthma symptoms, and reversibility in CCAAPS and MPAACH children by 2 GLI equations [file jamanetwopen-e2462176-s001.pdf]

Supplemental Online Content

Chang WC, Burkle JW, Williams LR, et al. Race-specific and race-neutral equations for lung function and asthma diagnosis in Black children. *JAMA Netw Open*. 2025;8(2):e2462176. doi:10.1001/jamanetworkopen.2024.62176

eMethods.
eReferences.
eTable. Model structures of 2 GLI equations
eFigure1. CONSORT diagram of the subjects included in the analyses
eFigure2. Changes in % FEV1/FVC using race-specific vs race-neutral equations
eFigure3. Estimated values of lung function measures using race-specific and race-neutral equations in CAMP
eFigure4. Estimated values of lung function measures using race-specific and race-neutral equations in CCAAPS
eFigure5. Estimated values of lung function measures using race-specific and race-neutral equations in MPAACH
eFigure6. Associations between % estimated lung function measures by GLI equations and African ancestry
eFigure7. Prevalence of asthma, asthma symptoms, and reversibility in CCAAPS and MPAACH children by 2 GLI equations

This supplemental material has been provided by the authors to give readers additional information about their work.

© 2025 Change WC et al. *JAMA Network Open*.

eMethods

Study Participants

The Childhood Asthma Management Program (CAMP)[1] is a multicenter, randomized clinical trial aimed at assessing the long-term effects of three inhaled medications for mild to moderate childhood asthma. 1041 children, aged 5-12 years, with mild-to-moderate current asthma and sensitivity to methacholine were enrolled between 1993 and 1995, with a follow-up period of 5 to 6 years. Each child's parent or guardian signed a consent statement approved by the clinic's institutional review board (IRB). CAMP data were downloaded with permission from the NIH Biologic Specimen and Data Repository Information Coordinating Center (https://biolincc.nhlbi.nih.gov/studies/camp/), accession number HLB00680815a.

The Cincinnati Childhood Allergy and Air Pollution Study (CCAAPS) cohort is a longitudinal atopic cohort that has been previously described.[2] Briefly, infants born in Cincinnati, Ohio, and Northern Kentucky between 2001 and 2003 with a parent with at least one allergy symptom and/or asthma and skin prick test (SPT) positive to at least one aeroallergen were eligible to participate. Participants were followed up until the age of 7. The study was approved by the IRB at the University of Cincinnati and all participants provided informed consent.

The Mechanisms of Progression from Atopic Dermatitis to Asthma in Children (MPAACH) is a prospective early-life cohort of children with AD that is designed to elucidate AD endotypes that progress to asthma. The MPAACH study design and methods have been detailed previously.[3,4] Briefly, children aged 0–2 years, had a gestation of ≥36 weeks, and either a diagnosis of AD (based on the Hanifin and Rajka Criteria for Atopic Dermatitis[5]) or the parent(s)/legal authorized representative indicates a positive response to each of the 3 questions from the Children's Eczema Questionnaire[6] were recruited between 2016 and 2024 and followed for 5 years across 5 annual visits, where pulmonary function test was administered at the fifth visit (V5). This study was approved by the Cincinnati Children's Hospital Medical Center IRB under protocol number 2016-5842, and informed consent was provided by parents or guardians of participants.

Pulmonary function testing

Enrolled CAMP participants completed spirometry at baseline and at each follow-up visit, at least 4 hours after the last use of a short-acting bronchodilator and at least 24 hours after the last use of theophylline or a long-acting bronchodilator. Spirometry was done before and after the administration of 2 puffs of albuterol, with a 15-minute interval in between.[1] The testing protocol followed ATS standards.[7]

In CCAAPS, all children completed spirometry at age 7 (Koko; nSpire Health, Longmont, Colo) according to the ATS criteria and calibration was performed daily. In MPAACH, spirometry was performed on a handled spirometer (CareFusion, Hoechberg, Germany, 2023) by children aged 7-8 years at V5, with calibration applied per ATS guidelines prior to testing. Flow-volume maneuver was visualized by software (SentrySuite, Vyaire, 2023) to assess satisfaction of the test requirements. Information of participants including date of birth, sex, standing height, weight and self-reported race was entered into the software. Standardized instructions and practices on performing forced expiratory maneuvers were given to the participants immediately before testing. Spirometry was performed in a standing position with a nose clip while under the supervision of the clinical staff. Each maneuver was assessed by clinical staff using ATS criteria and ATS preschool guidelines[8,9] for acceptability and repeatability. The criteria for satisfying an acceptable test included an indication of a rapid onset exhalation, a clear cycle of inhalation and exhalation, high or clearly determined initial peak flow with no cough or glottic closure, no early termination, and little to no back extrapolation. In addition, 1 second of forced expiratory time (FET) was required for children aged 7 years and 6 seconds of FET or 1 full second of an exhalation plateau was required for children aged 8 years. A minimum of 3 maneuvers were carried out by each child. Testing was terminated if a child produced 3 acceptable and 2 repeatable maneuvers or did not meet the criteria for satisfaction after 8 attempts. In CCAAPS, children who reported asthma symptoms, had an exhaled nitric oxide concentration >20 ppb, or a % predicted FEV1 <90% and/or %FEV1/FVC less than the lower limit of normal were eligible for post-bronchodilator testing. In MPAACH, children who met the acceptability criteria during pre-bronchodilator testing were sent for post-bronchodilator testing. Children were given 2 puffs of bronchodilator 15 minutes before performing post-bronchodilator testing. All clinical staff in the study underwent training to obtain optimal spirometry results. Spirometry results were reviewed by physicians, and trials with the best forced expiratory volume in the first second (FEV1) and forced vital capacity (FVC) were determined.

© 2025 Change WC et al. *JAMA Network Open*.

Eligibility for post-reversibility testing and asthma diagnosis in CCAAPS

After completing baseline spirometry, a subset of CCAAPS children were sent for post-bronchodilator testing if one of the following criteria were met: (1) parental report of asthma symptoms in the previous 12 months (tight or clogged chest or throat in the past 12 months, difficulty breathing or wheezy after exercise, wheezing or whistling in the chest in the previous 12 months, or a previous doctor's diagnosis of asthma); (2) predicted FEV1 of less than 90% (based on the race-specific equation); or (3) exhaled nitric oxide level of 20 ppb or greater.24 Children with <12% reversibility were further evaluated by methacholine challenge testing. Children were defined as having asthma if the parent reported asthma symptoms (as previously defined) and the child demonstrated either significant airway reversibility (≥12% increase in FEV1) or a positive methacholine challenge.

Asthma diagnosis in MPAACH

At age 7-8, all children in MPAACH undergo spirometry and reversibility testing. Children are defined as having asthma if the parent reports ≥1 wheezing symptom or ≥1 doctor or emergency department visit for asthma in the last 12 months AND the child demonstrates ≥8% reversibility. Asthma is also defined if the parent reports any of the following in the last 12 months: 1) ≥2 wheezing symptoms that last ≥24 hours and are separated by ≥5 consecutive days of no wheeze; 2) ≥1 hospitalization for asthma; 3) ≥2 doctor or emergency department visits for asthma; 4) ≥6 months of asthma controller use.

Race and Ancestry

In CAMP, parental reports of race/ethnicity were collected as White, Black, Hispanic, or Other at baseline. In CCAAPS, parent-reported race was collected as American Indian/Alaskan Native, Asian, Black/African-American, Pacific Islander, Other, White, and more than one race. In MPAACH, race was parent-reported via questionnaire upon enrollment and at each visit. Options were American Indian/Alaskan Native, Asian, Black/African American, Native Hawaiian/Pacific Islander, White, Other, Don't know, and choosing more than one option was allowed. 192 (18%) out of 1041 CAMP, 39 (6%) out of 617 CCAAPS participants, and 11 (9%) out of 117 MPAACH were self-reported as other race or mixed-race and were excluded from the analysis.

430 ancestry informative markers (AIMs) in CCAAPS and 592 AIMs in MPAACH from the Infinium Multi-Ethnic Global-8 1.0 BeadChip (Illumina, San Diego, CA) were used to infer population structure as previously described.[3] For this study, percent African Ancestry from the array results was categorized based on tertile and individuals were characterized into three groups – low, medium, and high.

Reference equations

The goal of the study was to compare spirometry results estimated by two equations established by The Global Lung Function Initiative (GLI) – 2012 GLI race-specific reference equation,[10] and 2022 GLI Global race-neutral reference equations.[11] Spirometry data used to construct the 2012 GLI reference equations included 74,187 individuals aged 2.5 to 95 years, who were non-smokers and were free from respiratory symptoms. Data was collected from 33 countries, comprising different ethnic groups. The equations were developed using the LMS method which modeled the age-varying distribution of the measurement in the expected mean, coefficient of variation and skewness.[12] The predicted values of FEV1, FVC, percent FVC exhaled in the first second (FEV1/FVC), and other indices were functions of sex, age, standing height and race/ethnicity including White, African American, South East Asians, North East Asians and for other ethnic groups or people of mixed descent **(eTable1)**. The 2022 GLI race-neutral reference equations were developed by reanalyzing the same set of individuals used in deriving 2012 GLI reference equations, with additional inverse probability weighting applied to reduce the bias associated with the over-representing of White individuals. The new equations removed the racial component from the model and estimated predicted values of lung function measures based only on sex, age, and standing height. In our analysis, self-reported race of each individual was entered into race-specific equations and racial factor was eliminated for race-neutral equations (**eTable1**).

Statistical analyses

Data were assessed and analyzed from November 15, 2023 to May 21, 2024. As the goal of the study was to assess lung function measurements using two GLI approaches, we included pre-bronchodilator results only from children who produced maneuvers that met all ATS criteria for acceptability and repeatability. R package *rspiro*,[13] which implemented multiple spirometry equations, was used to estimate predicted values of FEV1, FVC, and

© 2025 Change WC et al. *JAMA Network Open*.

FEV1/FVC of lung function measurements for both equations. The % predicted FEV1 was computed by dividing measured FEV1 from the best trial by the expected values of FEV1. The same method was applied to FVC and FEV1/FVC. Prior to the analysis, Shapiro-Wilk tests were performed to examine the distribution of the data for each continuous variable. Descriptive statistics are presented in median (25th-75th percentile) or frequency (%) due to the non-normal distributions. Comparisons between White and Black children were conducted using Wilcoxon rank-sum or chi-square tests for continuous and categorical variables, respectively. Paired Wilcoxon rank-sum tests were used to compare the median percent of predicted lung function values between each equation. Kruskal-Wallis test was used to compare lung function values across three African ancestry groups, followed by Benjamini-Hochberg adjustment where significance was observed. To determine the differences between each equation in the number of children with asthma symptoms identified, McNemar's test was used. Statistical significance was defined as p-value<0.05. Statistical analyses were performed in R version 4.1.0 (R core team, 2022).

© 2025 Change WC et al. *JAMA Network Open*.

eReferences

1. The Childhood Asthma Management Program (CAMP): design, rationale, and methods. Childhood Asthma Management Program Research Group. *Control Clin Trials.* 1999;20(1):91-120.
2. Brunst KJ, Ryan PH, Lockey JE, et al. Unraveling the relationship between aeroallergen sensitization, gender, second-hand smoke exposure, and impaired lung function. *Pediatr Allergy Immunol.* 2012;23(5):479-487.
3. Biagini JM, Kroner JW, Baatyrbek Kyzy A, et al. Longitudinal atopic dermatitis endotypes: An atopic march paradigm that includes Black children. *J Allergy Clin Immunol.* 2022;149(5):1702-1710 e1704.
4. Biagini Myers JM, Sherenian MG, Baatyrbek Kyzy A, et al. Events in Normal Skin Promote Early-Life Atopic Dermatitis-The MPAACH Cohort. *J Allergy Clin Immunol Pract.* 2020;8(7):2285-2293 e2286.
5. Hanifin JM RG. Diagnostic features of atopic dermatitis. . 1980;92(44):7.
6. von Kobyletzki LB, Berner A, Carlstedt F, Hasselgren M, Bornehag CG, Svensson A. Validation of a parental questionnaire to identify atopic dermatitis in a population-based sample of children up to 2 years of age. *Dermatology.* 2013;226(3):222-226.
7. Miller MR, Hankinson J, Brusasco V, et al. Standardisation of spirometry. *Eur Respir J.* 2005;26(2):319-338.
8. Gaffin JM, Shotola NL, Martin TR, Phipatanakul W. Clinically useful spirometry in preschool-aged children: evaluation of the 2007 American Thoracic Society Guidelines. *J Asthma.* 2010;47(7):762-767.
9. Miller MR, Crapo R, Hankinson J, et al. General considerations for lung function testing. *Eur Respir J.* 2005;26(1):153-161.
10. Quanjer PH, Stanojevic S, Cole TJ, et al. Multi-ethnic reference values for spirometry for the 3-95-yr age range: the global lung function 2012 equations. *Eur Respir J.* 2012;40(6):1324-1343.
11. Bowerman C, Bhakta NR, Brazzale D, et al. A Race-neutral Approach to the Interpretation of Lung Function Measurements. *Am J Respir Crit Care Med.* 2023;207(6):768-774.
12. Cole TJ. The LMS method for constructing normalized growth standards. *Eur J Clin Nutr.* 1990;44(1):45-60.
13. Lytras T. **rspiro: Implementation of Spirometry Equations. R package version 0.4.** 2023.

© 2025 Change WC et al. *JAMA Network Open*.

eTable. Model structures of 2 GLI equations

Equations	Factors included	General model structure[a]
The 2012 GLI race-specific reference	1. Sex[b]<br>2. Standing height<br>3. Age<br>4. Race/ethnicity (White [baseline], African American, South East Asians, North East Asians and for other/mixed ethnic groups. Assign specific race to participants based on self-reported race)	Predicted value = exp(a0 + a1*ln(Height) + a2*ln(Age) + a3*AfrAm + a4*NEAsia + a5*SEAsia + a6*Other + Mspline)
The 2022 GLI race-neutral reference	1. Sex[b]<br>2. Standing height<br>3. Age	Predicted value = exp(a0+ a1*ln(Height) + a2*ln(Age) + Mspline)

[a] AfrAM, African American; NEAsia; North East Asians; SEAsia, South East Asians; Other, other/mixed ethnic groups.
[b] Two models with the same structure but different coefficients were developed for male and female, respectively.

© 2025 Change WC et al. *JAMA Network Open*.

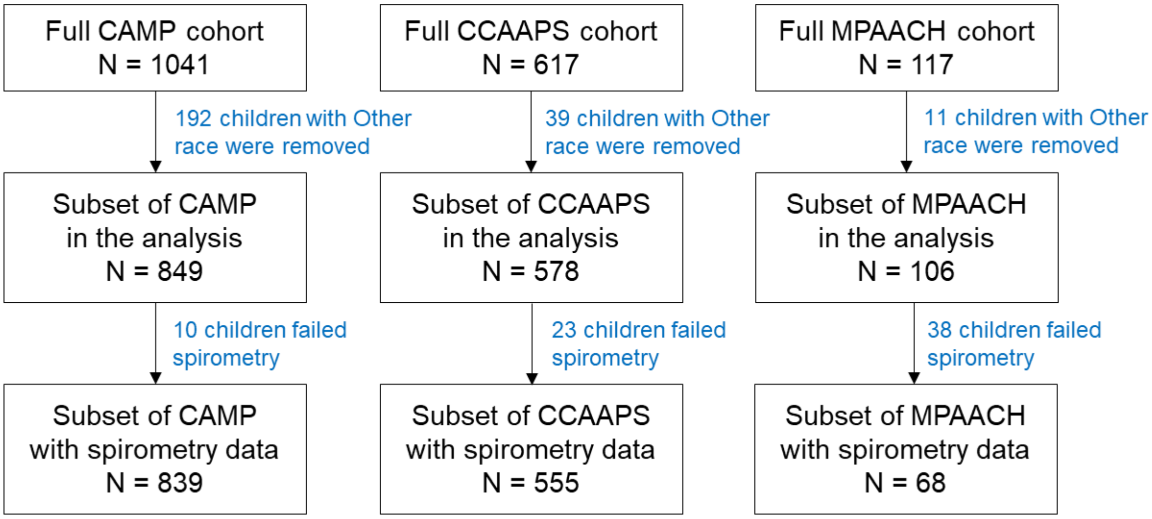

eFigure1. CONSORT diagram of the subjects included in the analyses.

© 2025 Change WC et al. *JAMA Network Open*.

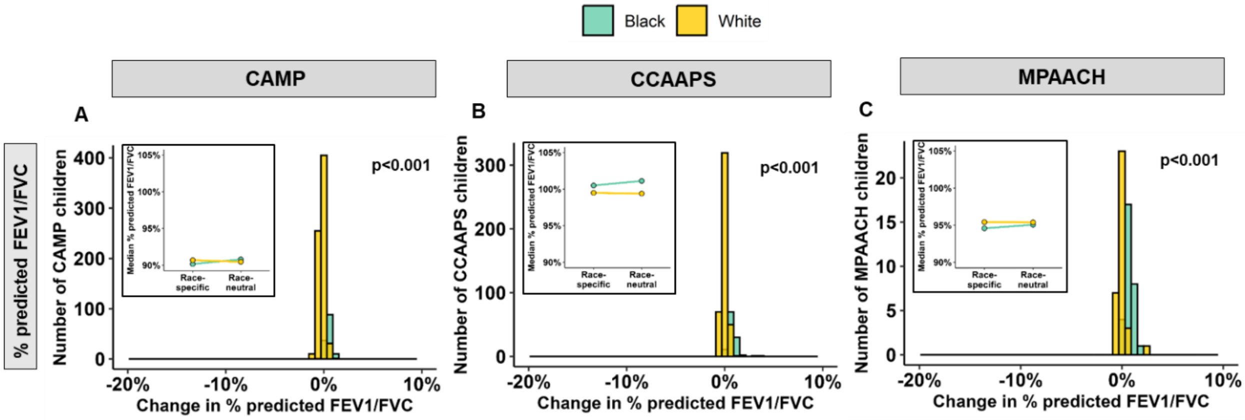

eFigure2. Changes in % FEV1/FVC using race-specific vs race-neutral equations in black (green) and white (yellow) children. Differences in % predicted FEV1/FVC among **(A)** CAMP children (Black n = 137, White n = 702), **(B)** CCAAPS children (Black n = 113, White n = 442), and **(C)** MPAACH children (Black n = 31, White n = 37) when applying race-specific and race-neutral equations. An increase in predicted values represented an improvement in apparent pulmonary function, whereas a decrease indicated a decline in apparent pulmonary function. Changes in the predicted values were calculated as the % predicted derived from race-neutral equation minused the % predicted derived from race-specific equation. Differences between races were compared using Wilcoxon rank-sum tests.

© 2025 Change WC et al. *JAMA Network Open*.

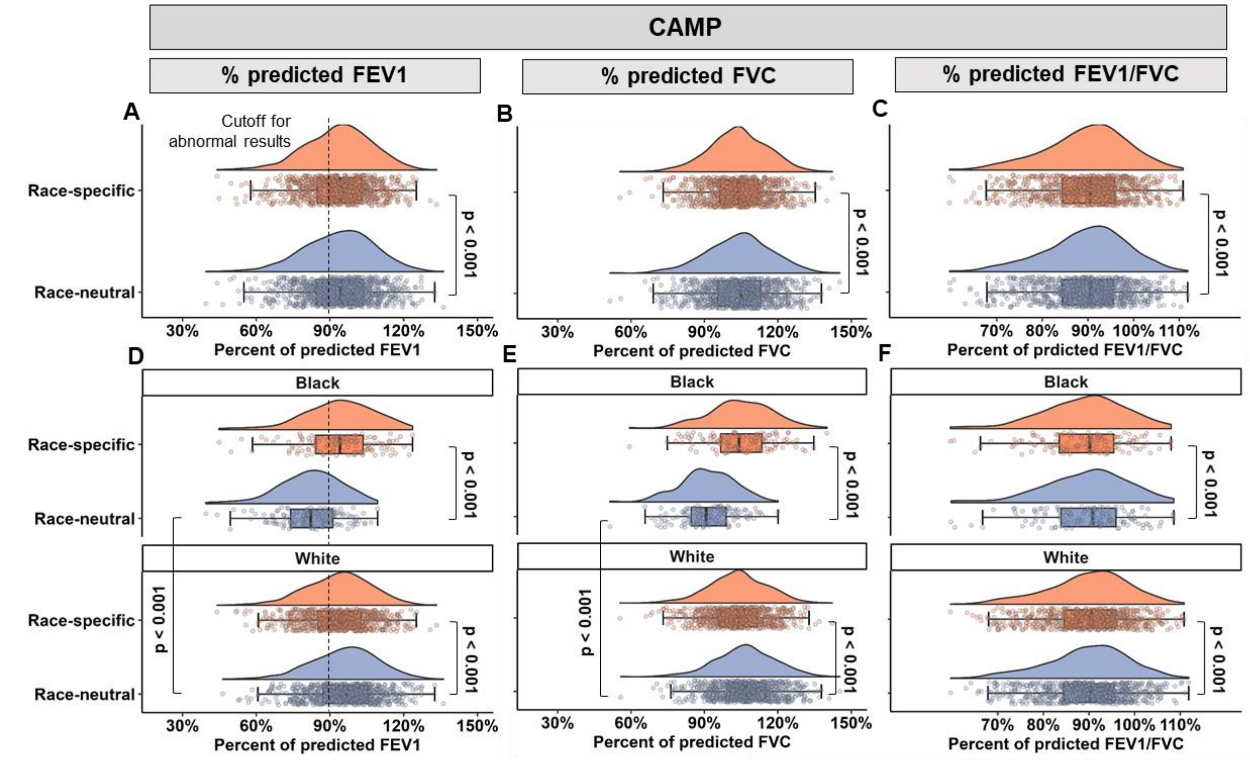

eFigure3. Estimated values of lung function measures using race-specific (orange) and race-neutral (purple) approaches in CAMP. % predicted **(A)** FEV1, **(B)** FVC, and **(C)** FEV1/FVC for overall CAMP children calculated based on the two GLI equations. **(D-F)** Results were stratified by self-reported race. The percent of predicted values between the two equations were compared using paired Wilcoxon rank-sum tests for overall cohort and within each racial group. Wilcoxon rank-sum tests were used when comparing between Black and White children. Dashed lines represent cut-off for <90%predictedFEV1.

© 2025 Change WC et al. *JAMA Network Open*.

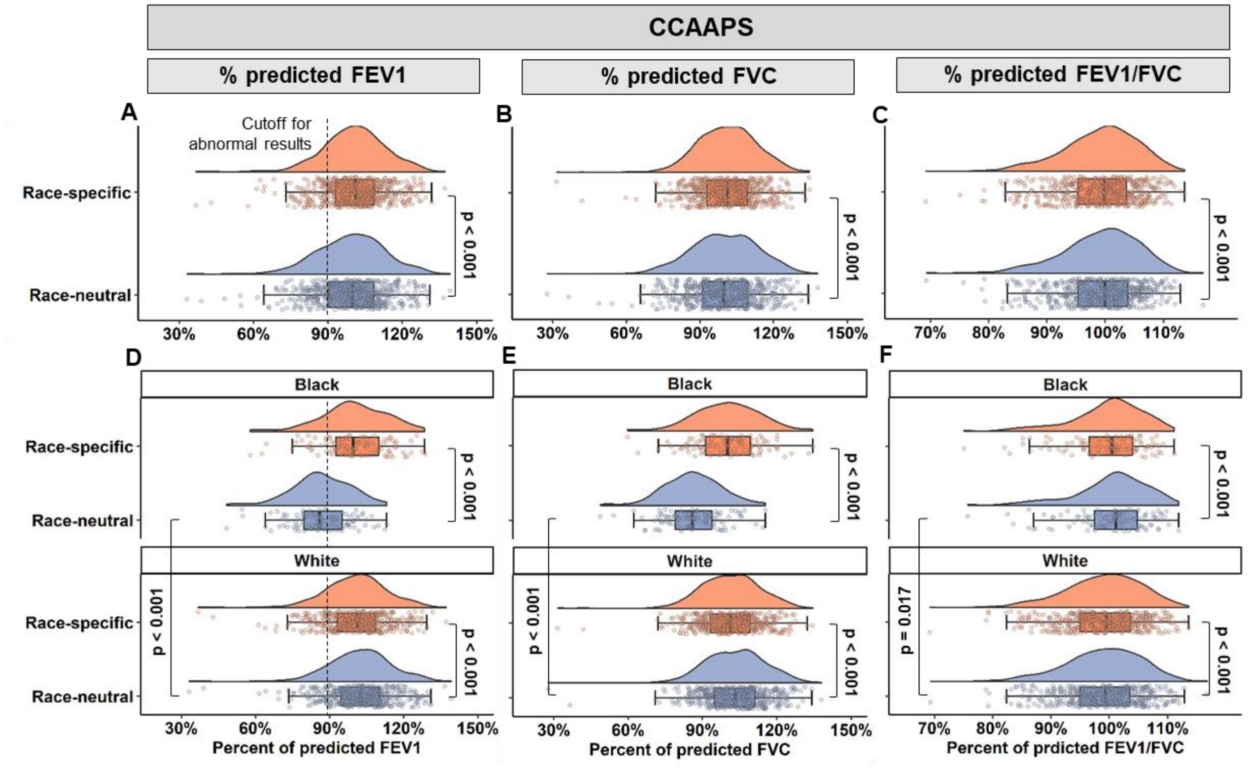

eFigure4. Estimated values of lung function measures estimated using race-specific (orange) and race-neutral (purple) approaches in CCAAPS. % predicted **(A)** FEV1, **(B)** FVC, and **(C)** FEV1/FVC for overall CCAAPS children calculated based on the two GLI equations. **(D-F)** Results were stratified by self-reported race. The percent of predicted values between the two equations were compared using paired Wilcoxon rank-sum tests for overall cohort and within each racial group. Wilcoxon rank-sum tests were used when comparing between Black and White children. Dashed lines represent cut-off for <90%predictedFEV1.

© 2025 Change WC et al. *JAMA Network Open*.

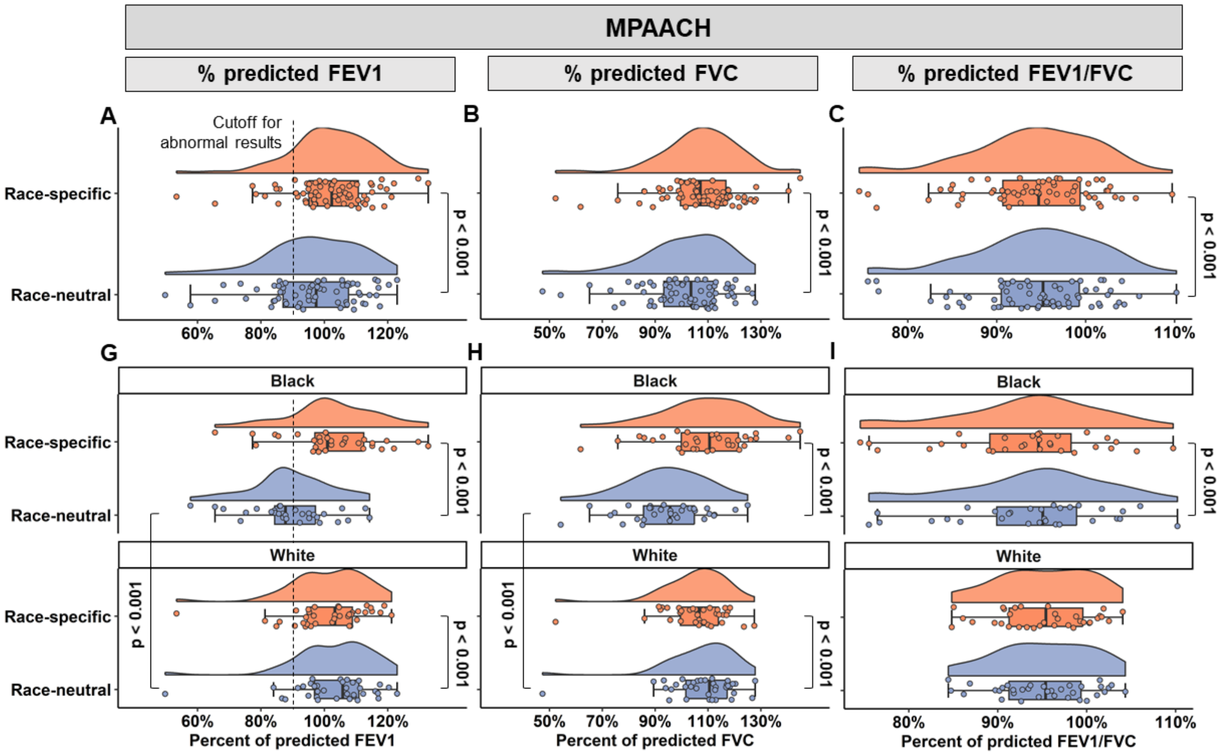

eFigure5. Estimated values of lung function measures estimated using race-specific (orange) and race-neutral (purple) approaches in MPAACH. % predicted **(A)** FEV1, **(B)** FVC, and **(C)** FEV1/FVC for overall MPAACH children calculated based on the two GLI equations. **(D-F)** Results were stratified by self-reported race. The percent of predicted values between the two equations were compared using paired Wilcoxon rank-sum tests for overall cohort and within each racial group. Wilcoxon rank-sum tests were used when comparing between Black and White children. Dashed lines represent cut-off <90%predictedFEV1.

© 2025 Change WC et al. *JAMA Network Open*.

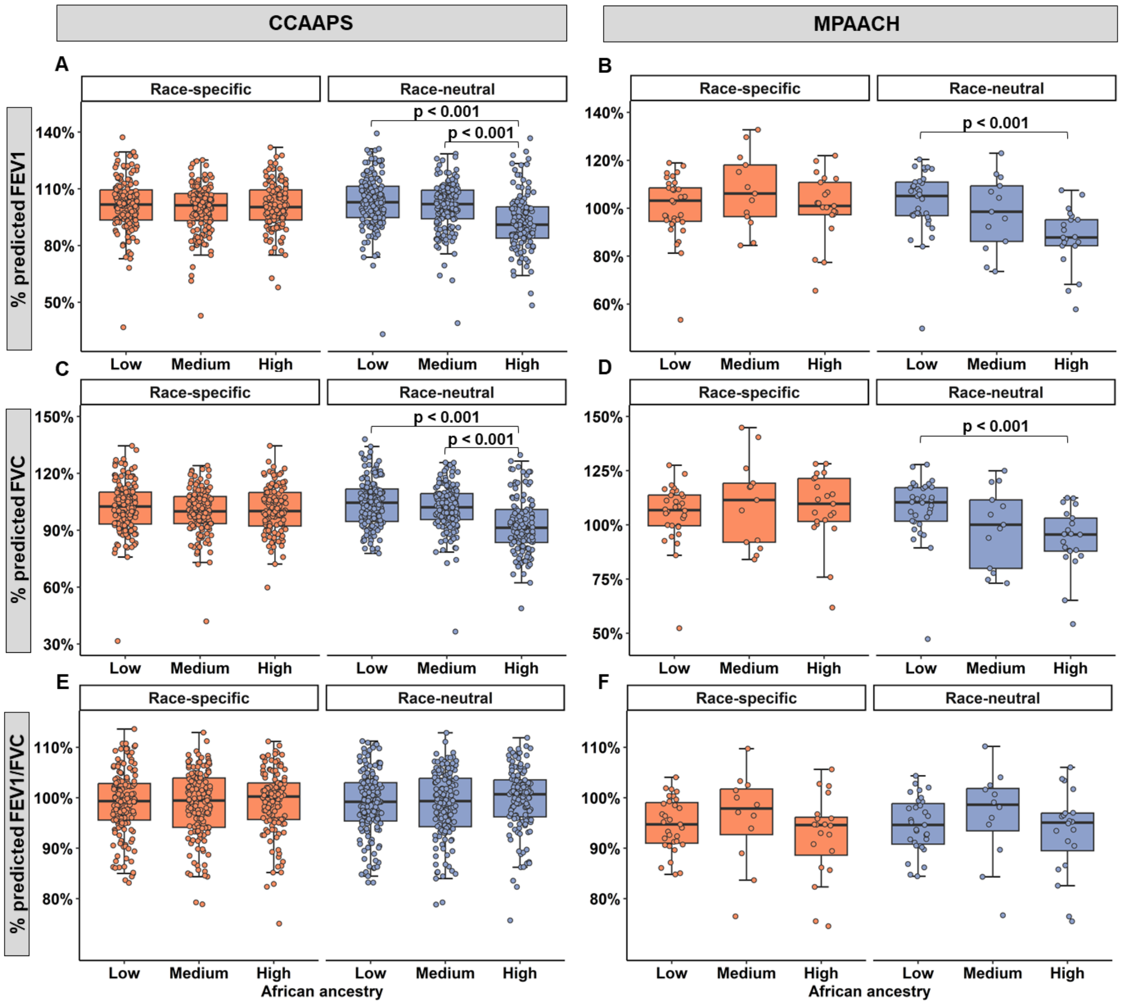

eFigure6. Associations between % estimated lung function measures by GLI equations and African ancestry. % predicted **(A-B)** FEV1, **(C-D)** FVC, and **(E-F)** FEV1/FVC in children with low, medium, and high African ancestry in CCAAPS and MPAACH. Percent African Ancestry were categorized based on tertile. Comparisons across three ancestry groups were done using Kruskal-Wallis tests, followed by Dunn's tests with Benjamini-Hochberg adjustment where significance was observed. The percent of predicted values was assessed using race-specific (orange) and race-neutral (purple) equations.

© 2025 Change WC et al. *JAMA Network Open*.

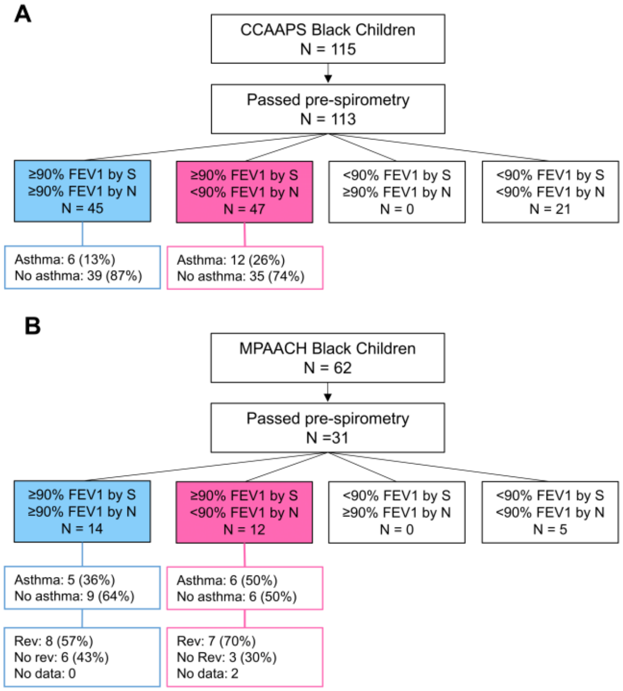

eFigure7. Prevalence of asthma, asthma symptoms, and reversibility in CCAAPS and MPAACH children by 2 GLI equations. Among children with a %predicted FEV1 of ≥90% using race-specific equations, the prevalence of asthma diagnosis, clinical symptoms of asthma, and reversibility were broken into two sub-groups: (1) remained with a ≥90% predicted FEV1 based on race-neutral equations (blue) or (2) switched to a <90% predicted FEV1 when based on race-neutral equations (pink). Number (%) of children with asthma, asthma symptoms, and reversibility in **(A)** Black CCAAPS, **(B)** Black MPAACH, **(C)** White CCAAPS, and **(D)** White MPAACH children. S, race-specific equations; N, race-neutral equations; Rev, reversibility.

© 2025 Change WC et al. *JAMA Network Open*.
