# Supplementary material for: Race-Specific and Race-Neutral Equations for Lung Function and Asthma Diagnosis in Black Children
Source: JAMA Netw Open. 2025 Feb 28;8(2):e2462176. doi: 10.1001/jamanetworkopen.2024.62176 (PMC11871546; doi:10.1001/jamanetworkopen.2024.62176)
Supplement: Supplement 2. — Data Sharing Statement [file jamanetwopen-e2462176-s002.pdf]

Data Sharing Statement

Chang. Use of Race-Specific and Race-Neutral Equations for Lung Function and Asthma Diagnosis in Black Children. *JAMA Netw Open*. Published February 28, 2025. doi:10.1001/jamanetworkopen.2024.62176

Data
Data available: No

Additional Information
Explanation for why data not available: Data will be made available upon request.
